# Glucocorticoid resistance conferring mutation in the C-terminus of GR alters the receptor conformational dynamics

**DOI:** 10.1038/s41598-021-92039-9

**Published:** 2021-06-15

**Authors:** Anna Kaziales, Florian Rührnößl, Klaus Richter

**Affiliations:** Department of Chemistry, Center for Integrated Protein Science Munich, Technische Universität München, Lichtenbergstr. 4, 85748 Garching, Germany

**Keywords:** Biochemistry, Biophysics, Biotechnology

## Abstract

The glucocorticoid receptor is a key regulator of essential physiological processes, which under the control of the Hsp90 chaperone machinery, binds to steroid hormones and steroid-like molecules and in a rather complicated and elusive response, regulates a set of glucocorticoid responsive genes. We here examine a human glucocorticoid receptor variant, harboring a point mutation in the last C-terminal residues, L773P, that was associated to Primary Generalized Glucocorticoid Resistance, a condition originating from decreased affinity to hormone, impairing one or multiple aspects of GR action. Using in vitro and in silico methods, we assign the conformational consequences of this mutation to particular GR elements and report on the altered receptor properties regarding its binding to dexamethasone, a NCOA-2 coactivator-derived peptide, DNA, and importantly, its interaction with the chaperone machinery of Hsp90.

## Introduction

Glucocorticoid receptor (GR) is a conserved steroid hormone receptor (SHR) and as such, belongs to the nuclear receptor family of ligand-activated transcription factors^1,2^. Glucocorticoids (GCs) are some of the most pervasive messengers and GR is expressed in nearly all cells, regulating a plethora of functions in health and disease^3^. GR regulates essential physiological processes such as development, reproduction, metabolism and homeostasis, and also many functions of the central nervous system, such as cognition, mood and sleep^1–4^.

Like many signaling proteins, mammalian SHRs are dependent on the chaperone machinery of heat-shock protein 90 (Hsp90) to acquire their active conformation^5–9^. Hsp90 is a highly conserved and abundant molecular chaperone that acts as the central hub of proteome homeostasis, proteostasis, and is involved in a broad spectrum of cell functions, including signal transduction, cellular trafficking, chromatin remodeling, cell growth, differentiation, and reproduction^6,10,11^. It supports folding, maturation and degradation of its client proteins by performing a dynamic, nucleotide-induced conformational cycle^5,6,12–14^. Unlike molecular chaperones, such as Hsp70, that bind unfolded/ nascent polypeptides, Hsp90, with the assistance of several co-chaperones, maintains client proteins in a nearly folded conformation poised to respond to an activation signal, such as hormone binding^15^. In the absence of ligand, GR is part of a multi-protein complex containing Hsp90 and other Hsp90 co-chaperones^7–9,16–18^. After participating in a series of chaperone assemblies and binding to ligand, a hyper-phosphorylated and transcriptionally active GR, is transported to the nucleus^2,19^. It then homo- or heterodimerizes, recruits coactivators/corepressors, and other transcription factors, binds to glucocorticoid response elements (GREs) and modulates many distinct gene networks^2,3,20,21^. Moreover, the ligand-activated, membrane-bound GR is reported to mediate non-genomic events, such as triggering the activation of kinase signaling cascades, including the mitogen.-activated protein kinase (MAPK) or the phosphatidylinositol 3-kinase (PI3K) pathways^22^. Even though much progress has been done on comprehending the Hsp90-client interplay, with steroid receptors playing a key role in such studies, as obligate Hsp90 clients, the molecular mechanism of this interaction is still enigmatic and the actual transformations on GR are yet to be clarified^14,23–25^.

Primary Generalized Glucocorticoid Resistance (PGGR) or Chrousos syndrome is a condition characterized by generalized, partial tissue insensitivity to glucocorticoids^21,26,27^. Patients with PGGR have defective GC negative feedback loops, which lead to compensatory hyperactivation of the HPA axis^21^. The elevated plasma adrenocorticotropic hormone (ACTH) causes adrenal hyperplasia and increased production of steroid precursors. The molecular basis of this condition is attributed to mutations in the human glucocorticoid receptor-α gene (*NR3C1*), which impair one or multiple aspects of GR action^21,27^. The impairment originates from the decreased affinity to hormone and thus, alters tissue sensitivity to GCs^21,27^. To date, 24 different GC resistant mutants have emerged in the clinic, in the DBD or the LBD of GR, causing a broad spectrum of clinical manifestations of variable severity^21,27–32^. Symptoms, when PGGR is pronounced, are relevant to mineralocorticoid and/or androgen excess, for instance hypofertility, hypertension, hypokalemic alkalosis, as well as anxiety and depression^26^. In addition, some patients with GC resistance do not harbor a mutation in the *NR3C1* gene, which implies that other factors participating in GC signal transduction, might as well be responsible for impaired GC sensitivity^33^. Among the various reported mutations, we here selected the case, where the disease was caused by a heterozygous mutation (T → C) at nucleotide position 2318 (exon 9) of the hGRα gene, which results in substitution of leucine by proline at amino acid position 773 in GR’s C-terminus^32^. The affected young woman reported fatigue, anxiety and was diagnosed with hyperandrogenism and hypertension^32^. Mutations of the last 14 residues cause alterations in hormone binding specificity and agonist potential while C-terminal deletions, yield inactive receptors^34^. This conserved region is required for ligand binding; however, the differential hormone-binding capacities of SHRs are not encoded in this region^34^. Charamandari et al. report that the L773P mutant demonstrates decreased transcriptional activity, decreased affinity for ligand, delayed nuclear translocation, altered interaction with the GRIP1/NCOA-2 coactivator, and exerts a dominant negative effect on wild-type hGRα^32^. Intrigued to understand the importance of GR’s C-terminus, we examined this mutant in vitro and in silico. We here identify the specific GR elements that exhibit altered conformational dynamics, and report on how this single point mutation affects the interaction with dexamethasone, DNA, an NCOA-2 peptide, and the chaperone machinery of Hsp90.

## Results

### The point mutation L773P does not have overt effects on the GRm protein

**Figure 1:**
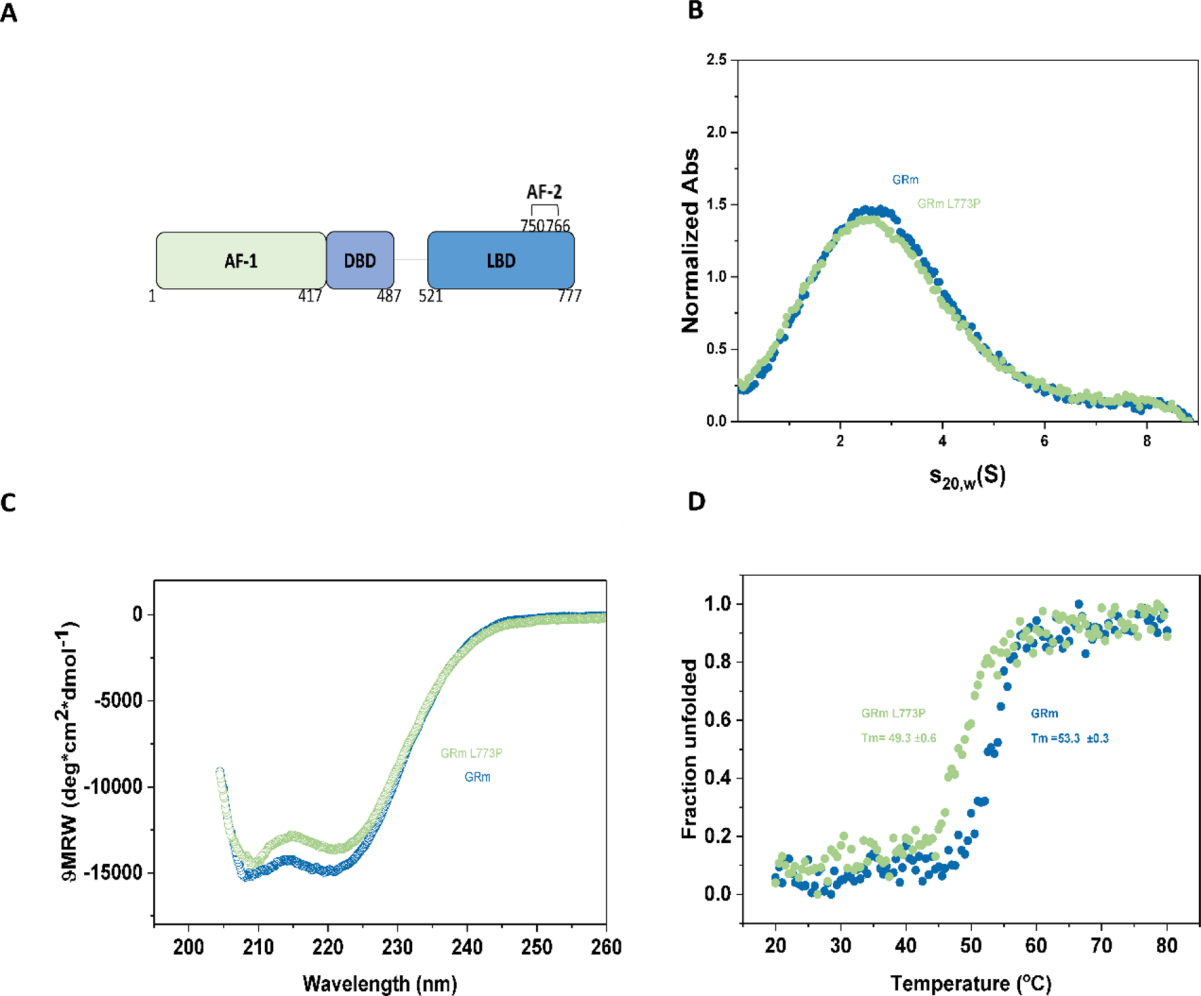
Single point mutation L773P does not have overt effects on GRm. **(A)** Schematic representation of GR domains. Protein constructs used in this study harbored the DBD, hinge and LBD domains of GR. **(B)** Sedimentation velocity AUC coupled to absorbance optics. Both constructs are monomeric. **(C)** Far-UV CD spectra of the two GRm variants show similar high α-helical content. **(D)** CD thermal transitions show that GRmL773P is less thermally stable, by approximately 3 °C.

GR shares the domain architecture that is conserved within the SHR family: an N-terminal transactivation domain (AF-1, aa 1–417), a zinc-finger DNA-binding domain (DBD, aa 418–487), a short hinge region (aa 488–520) and a ligand-binding domain (LBD, aa 521–777)(Fig. 1A)^35,36^. The GR LBD bound to dexamethasone (DEX) consists of in total 11 α-helices and 4 β-strands that form 2 short β-sheets^35,36^. SHR crystal structures with agonist ligands like DEX, have shown that the C-terminal α-helix, the activation function-2 (AF-2), serves in forming the lid of the ligand binding pocket^35,36^. Given that the wild type GR protein is rather unstable, we employ a GR variant, GRm, which is stabilized by mutagenesis and has previously been used to shed light on the GR•Hsp90 interaction^23–25^. To study the influence of the point mutation, we utilize protein constructs that contain the DBD, hinge region, and LBD of GR. Leucine 773 was mutated to proline and both proteins, GRm and GRm L773P, were purified from *E.coli* in the presence of the stabilizing ligand DEX. The two GR variants were initially characterized to see whether there are pronounced differences in the mutant receptor. Both receptor variants are monomeric and sediment with 2.8 S (Fig. 1B), as deteremined by analytical ultracentrifugation (AUC) coupled to absorbance detection. Far-UV circular dichroism spectroscopy displayed a similar, high α-helical content for the two variants (Fig. 1C). Both variants are stable up to 45 °C and then start unfolding in a cooperative transition, as determined by CD thermal transitions (Fig. 1D). The GRm L773P melting temperature was determined to be approximately 3 °C lower than that of GRm.

### GRm L773P exhibits reduced hormone binding that is restored by Hsp90β and altered binding to a coactivator-derived peptide

**Figure 2:**
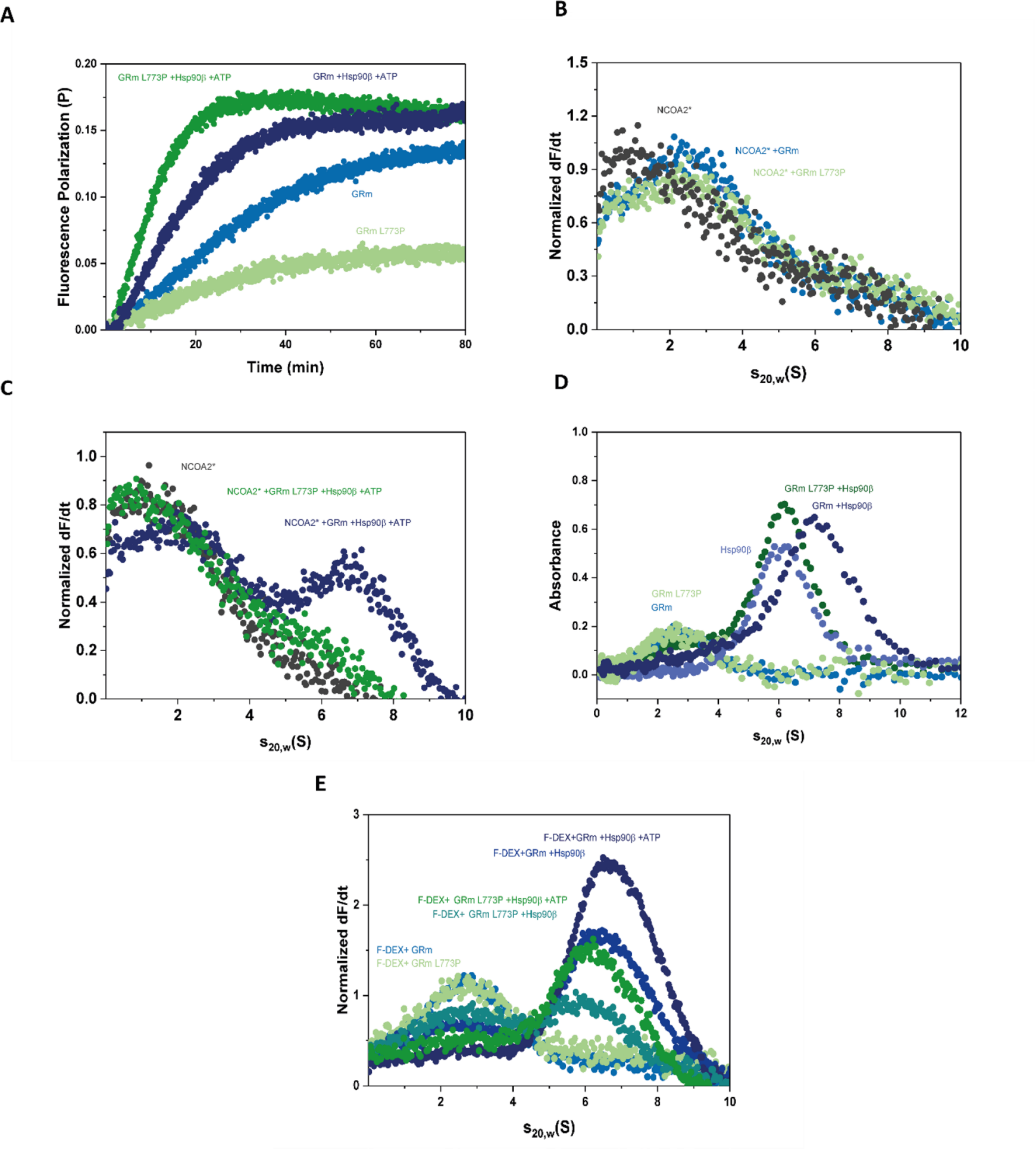
GRm L773P exhibits reduced hormone binding that is restored by Hsp90β and altered binding to a coactivator-derived peptide. **(A)** Fluorescence polarization kinetics show that Hsp90β is able to restore hormone binding to GRm L773P (olive). **(B,C)** Sedimentation velocity AUC coupled to fluorescence optics shows the binding of GRm and GRm L773P to a NCOA2-derived peptide in the presence and absence of Hsp90β. Only GRm is able to form a ternary complex with Hsp90β and the NCOA-2 peptide. **(D)** Sedimentation velocity AUC coupled to absorbance optics shows GRm and GRm L773P binding to Hsp90β in 1:1 stoichiometry in the absence of ATP. **(E)** Sedimentation velocity AUC following F-DEX shows that GRmL773P**∙**Hsp90β binds hormone to a lower extent than GRm, both in the absence and presence of ATP, but both GR·Hsp90β assemblies are competent in hormone-binding.

Although the exact molecular events are not yet clarified, Hsp90 is required to transform GR into a hormone-binding competent state in vivo. It is, however, known that purified GR is capable of ligand-binding in vitro in the absence of Hsp90^23–25^. To compare the two GRm variants, hormone-binding was followed by fluorescence polarization, recording GRm binding to fluorescein-labeled dexamethasone (F-DEX). Under these conditions, GRm L773P exhibited a 2.4-fold decrease of the apparent hormone-binding rate (Fig. 2A). Following GR binding to F-DEX in the presence of Hsp90β and ATP, we see that the chaperone can accelerate the reaction kinetics of GRm L773P to an extent, even greater than for the natural leucine GRm variant (Fig. 2A). Even though the apparent hormone binding rates may reflect exchange kinetics, since residual DEX may still be bound in the LBD despite the extensive dialysis of GRm, this data implies that interaction properties between GRm and Hsp90β may be modified by the mutation.

We then examined the interaction with a nuclear receptor coactivator 2-derived (NCOA2) peptide (residues 740 − 753), which binds via its LLXXLL motif to a pocket formed by AF-2, H3 and H4 of DEX-bound GR^35^. For this assay, we labeled the peptide with ATTO 488 and performed AUC coupled to fluorescence detection. The unbound NCOA-2 peptide, sediments with approximately 1 S and the complexes it is participating in, are observed at larger s-values. Both GRm variants could bind to the peptide, as observed at 2.8 S, with the L773P mutant exhibiting a slightly less efficient binding (Fig. 2B). However, when the chaperone Hsp90β is present, it is only possible for GRm to bind Hsp90β and the peptide simultaneously, in a complex sedimenting with an s_20,w_ of 7 S (Fig. 2C). In contrast, the L773P variant seems to be incapable of forming such ternary GR∙NCOA2∙Hsp90β complex (Fig. 2C).

### GRm L773P∙binds with reduced affinity to Hsp90β and Hsp90β-containing GRm L773P complexes are competent in hormone binding

To understand the differences in complex formation properties, the two GRm constructs were examined by sedimentation velocity AUC coupled to absorbance optics for their interaction with Hsp90β. Under these conditions, in 1:1 stoichiometry and absence of nucleotide that is crucial for hormone bindingGRm L773P barely binds the chaperone, compared to the GRm·Hsp90β complex that readily forms and can be observed at 7 S (Fig. 2D). These rather boundary conditions, exemplify the altered properties of the mutant receptor before proper interaction with Hsp90β/ATP.Then F-DEX was employed in AUC coupled to fluorescence optics, to assess the affinity of hormone-bound GR∙chaperone assemblies. Under these conditions, the fraction of monomeric GR that bound the ligand at 2.8 S was the same for both variants (Fig. 2E). Further, both variants can bind to Hsp90β, as observed by the respective peaks at 6.5 S and in both cases, ATP increases the hormone-bound GR∙Hsp90β population (Fig. 2E). Judging from the reduction of the monomeric GR peaks, observed at 2.8 S, GRmL773P∙Hsp90β complexes bound to hormone are represented by a lower population than for GRm both in the presence and absence of ATP. Nevertheless, presence of Hsp90β stimulates hormone binding in both cases, compared to monomeric GR, and in both cases, when ATP is added nearly all hormone-bound GR is in complex with Hsp90β.

### GRm L773P exhibits altered DNA binding properties

**Figure 3:**
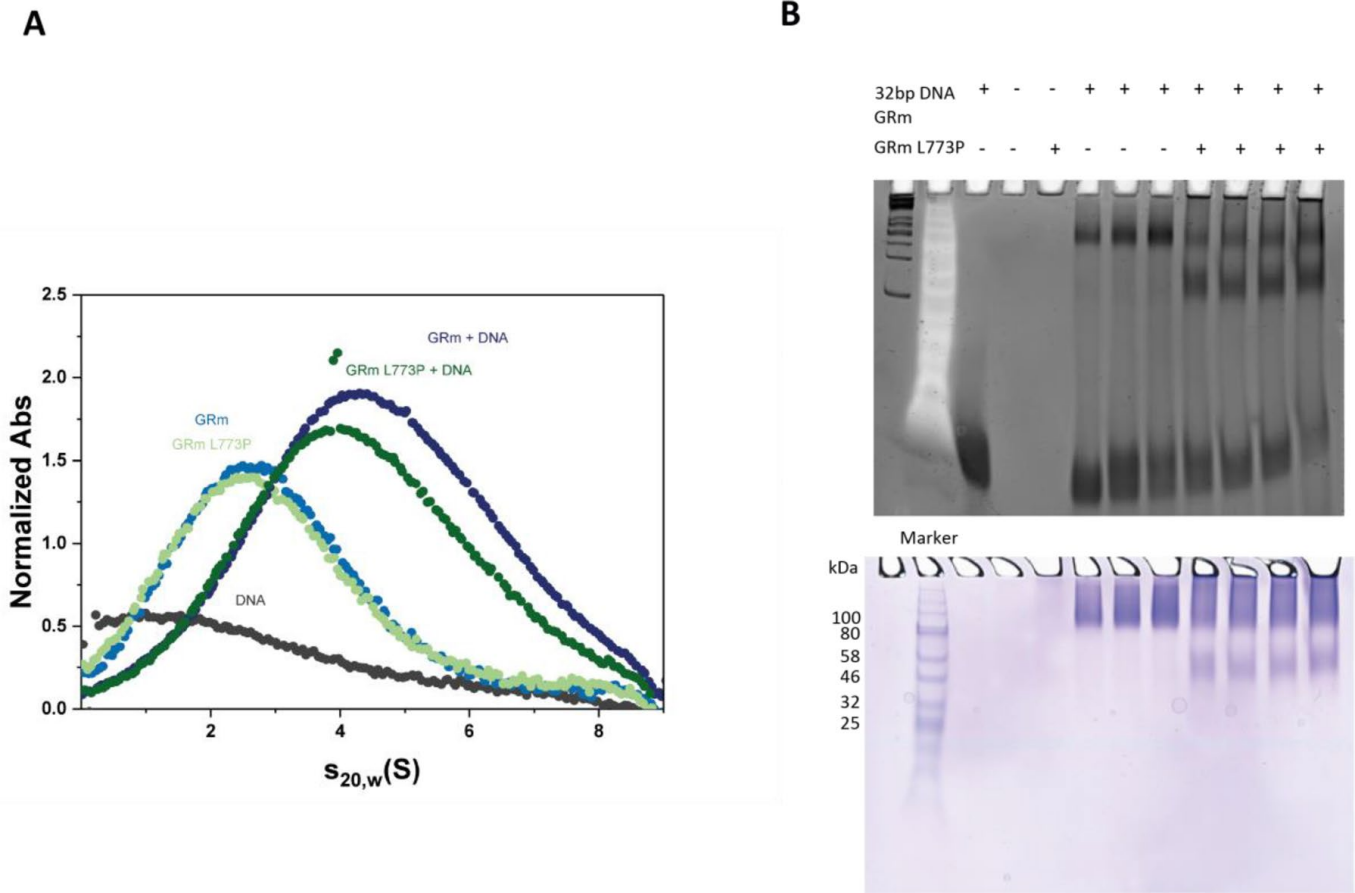
GRm L773P exhibits altered DNA binding properties. **(A)** Sedimentation velocity AUC experiment showing that both GRmL773P and GRm are competent in DNA binding. A slightly reduced s-value can be observed for GRmL773P + DNA. **(B)** EMSA assay under native conditions shows a high population of monomeric GRmL773P bound to DNA that is hardly visible for GRm, which forms dimers in the presence of DNA. Shown is a fluorescence scan that specifically detects DNA-containing bands and below a Coomasie stain to also detect protein species. Non-cropped gel images are presented in Supplementary Figure S6.

Having observed the differences in hormone and coactivator binding, the DNA binding competency of the two constructs was examined. A 32 bp GRE element from the *fkbp5* promoter was chosen, utilizing the eykaryotic promoter database, and was used for AUC experiments coupled to absorbance optics. This experiment shows that both constructs are able to bind to DNA, as shown by the peaks of species sedimenting at ~ 4S. However, the species observed for GRmL773P have a slightly reduced s-value (Fig. 3A). This implies either a lower affinity for the DNA or a different monomer: dimer ratio.

This was further examined with electrophoretic mobility assays (EMSA) under native conditions. GR without DNA was not able to enter in the gel under these conditions, but once DNA was added, the species formed could be separated by electrophoresis (Fig. 3B). It is clear from this assay that only GRm L773P forms a species matching the size of monomeric GR in complex with the 32 bp DNA (~ 50 kDa), in addition to a species matching the size of dimeric GRm in complex with DNA. This band is hardly observable for GRm, which at all concentrations tested, formed a species with the size of dimeric GR + DNA (Fig. 3B).

### Single point mutation L773P alters the dynamics of several GR elements in the presence and absence of the Hsp90β chaperone

To understand the complex interaction patterns that seem influenced by the single-point mutation, the two GRm variants were subjected to hydrogen/deuterium exchange coupled to mass spectrometry (H/DX-MS) in the presence of DEX, and the presence or absence of Hsp90β and ATP. Τhis analysis can reveal the conformational consequences of Hsp90β interaction with GRm while comparison to GRm L773P can point to the elements that make this GRm variant glucocorticoid resistant. High sequence coverage was obtained and by plotting the H/DX fractional uptake, both proteins seem highly dynamic, highlighting the ligand-induced plasticity of GR (Supplementary Figures S1, S2).

**Figure 4:**
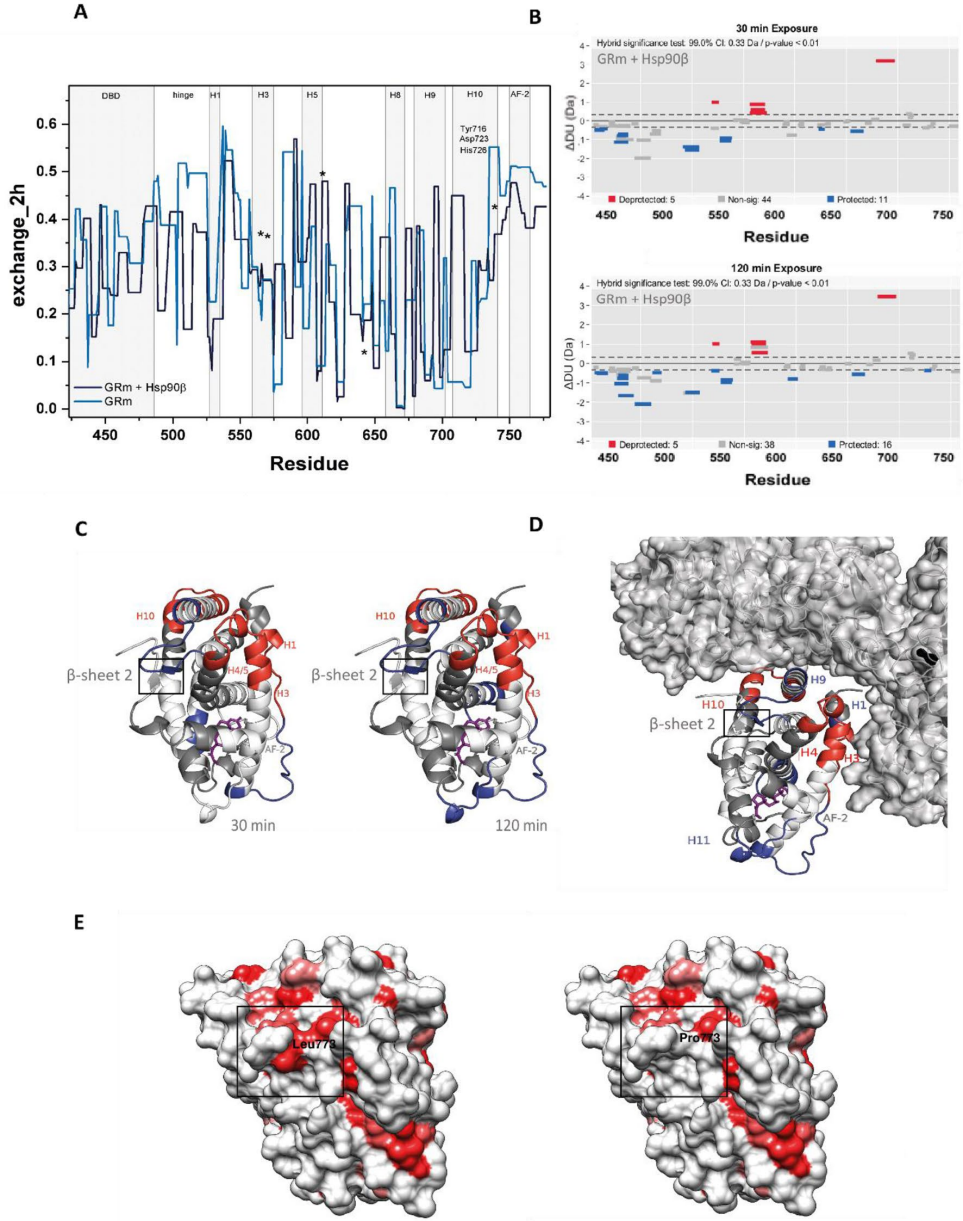
Comparison of GRm in the absence and in the presence of Hsp90β and ATP by hydrogen–deuterium exchange coupled to MS reveals the conformational consequences of chaperone binding to GRm. **(A)** Fractional uptake plots of GRm in the absence (blue) and presence (navy) of Hsp90β and ATP. Hormone binding residues are indicated with asterisks and characteristic GRLBD elements are highlighted on the plot. **(B)** Wood’s plots based on two experiments, constructed with the Deuteros software for time points of 30 and 120 min and using hybrid statistics (p-value < 0.01) show the significantly affected GR elements due to chaperone interaction. **(C)** Significant changes in fractional uptake are mapped on the GR LBD structure for 30 min and 2 h of exchange and are color coded in blue (low exchange) to red (high exchange). **(D)** Shown is the differential HD/X data for GRm in the presence of Hsp90β and ATP after 2 h of exchange, projected onto the GR·HSP-90 structure generated by crosslink-guided molecular docking. **(E)** GRLBD surface (PDB 5NFP) colored for amino acid hydrophobicity according to the Kyte-Doolittle scale. The hydrophobic residue network around the leucine 773 (left structure), as indicated with the frame is discontinued once leucine is mutated to proline (right structure).

We first examined GRm in the presence and absence of Hsp90β and ATP (Fig. 4A). Wood’s plots, constructed for different time points, indicate that GR elements are significantly affected by the chaperone interaction (Fig. 4B). This data is mapped on the GRLBD structure and helps visualize that β-sheet 2, consisting of the strand located between H8 and H9, and the C-terminal strand following AF-2, is significantly protected, when GR is in complex with Hsp90β (Fig. 4C). H9, H10 and H4/5 experience a mutual deprotection upon interaction with the chaperone while elements in H7, H4/5 and H3 exhibit lower fractional uptake (protection) around the ligand binding pocket. Mapping this data on the model we previously constructed for GRLBD in complex with nematode HSP-90 shows that these regions overlap with the receptor·chaperone interface,and the significant deprotection in the upper part of the receptor and around the protected β-sheet 2, may refelect the way the chaperone facilitates the access of ligand to the binding pocket (Fig. 4D)^25^. Interestingly, in the presence of chaperone the DBD, hinge, and H1 regions of GRm seem protected, implying a communication between the GRm domains during chaperone interaction.

**Figure 5:**
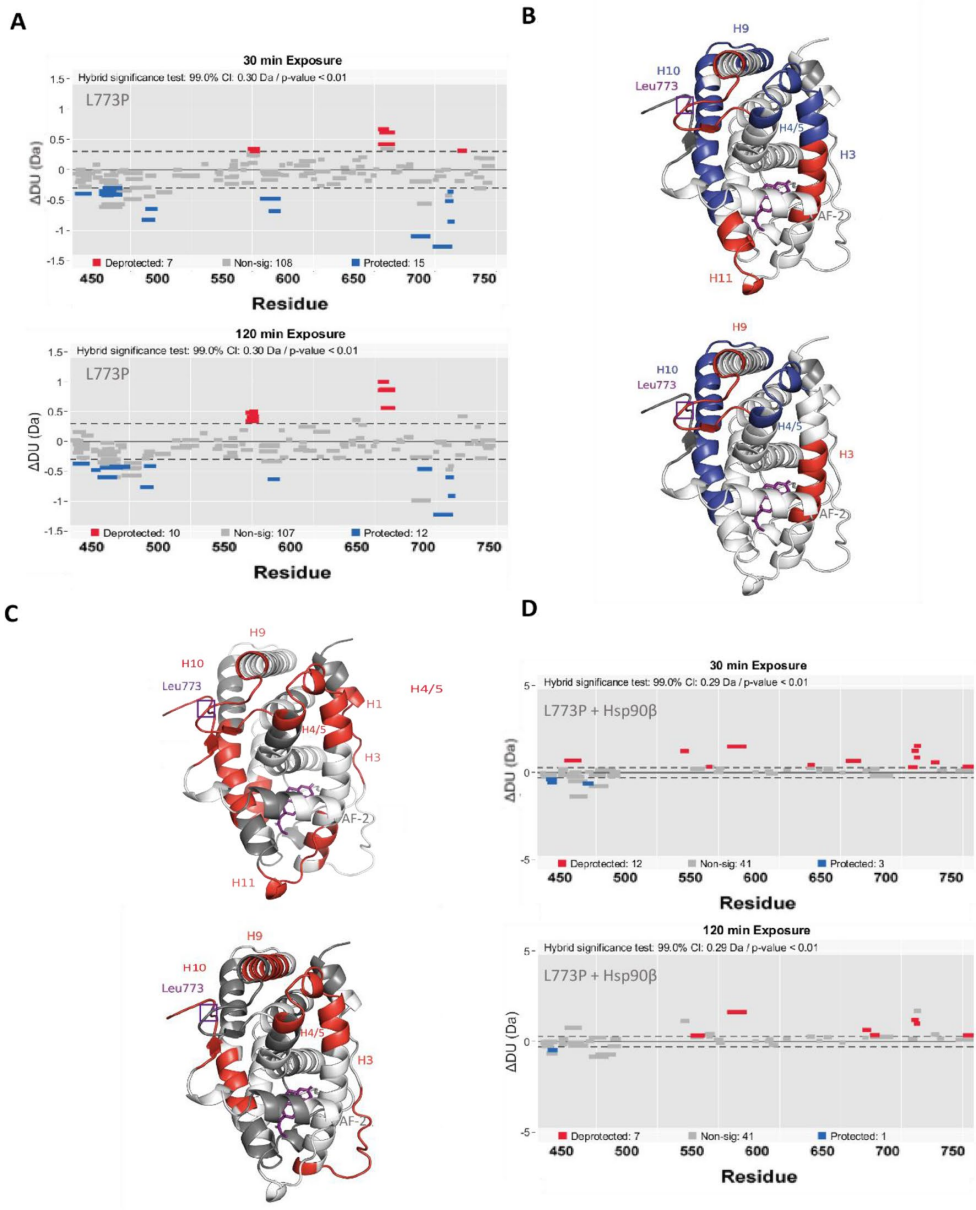
GRm L773P exhibits altered dynamics compared to GRm and in the presence of Hsp90β. **(A)** Wood’s plots based on two experiments, constructed with the Deuteros software for time points of 30 and 120 min and using hybrid statistics (p-value < 0.01). Significant differential signals concern the DBD/hinge, H3, H4/5, H9, H10 and β-sheet 2 of GR. **(B)** The most significant relative changes in fractional uptake between GRm and GRm L773P are mapped on the GR LBD structure for 30 min and 2 h of exchange and are color coded in blue (low exchange) to red (high exchange). **(C)** Significant changes in fractional uptake of GRm L773P in the presence of Hsp90β and ATP are mapped on the GR LBD structure for 30 min and 2 h and are color coded in blue (low exchange) to red (high exchange). **(D)** Wood’s plots based on two experiments, constructed with the Deuteros software for time points of 30 and 120 min and using hybrid statistics (p-value < 0.01) show the significantly affected GRm L773P elements in the presence of Hsp90β.

Then, GRm L773P was examined in the absence and presence of Hsp90β and ATP. Several GR elements exhibit altered dynamics in the mutant receptor, when compared to GRm (Fig. 5A). Wood’s plots, constructed for different time points, indicate that significant differences develop over time and affect H3, H4/5, H9 and H10 of GR LBD (Fig. 5A,B). Especially the proximal to the mutation site β-sheet 2, consisting of the strand located between H8 and H9 and the C-terminal strand following AF-2, exhibits higher fractional uptake (deprotection) in the mutant protein. Importantly, the DBD and hinge region of the receptor display altered dynamics, i.e. GRm L773P’s DBD exhibits significantly lower fractional uptake (protection) (Fig. 5A,B).

Comparing the H/DX of the mutant receptor in the absence and presence of chaperone, shows the effect that Hsp90β has on the mutant receptor, which displays an overall deprotection of the ligand binding pocket (Fig. 5C,D). In contrast to GRm, the β-sheet 2, is significantly deprotected upon interaction with Hsp90β (compared to the GRm analysis in Fig. 4B–D that was protected). The AF-2 interface and C-terminal peptide of GR harboring the mutation, show significant deprotection while no significantly protected peptides were observed around the ligand binding pocket. On the contrary, the elements from H7 and H11 that get protected upon interaction with Hsp90β in the case of GRm, here show a relative deprotection. The DBD and hinge regions of the mutant receptor also experience a deprotection that was not observed in the GRm analysis (Fig. 4B–E versus Fig. 5C,D).

**Figure 6:**
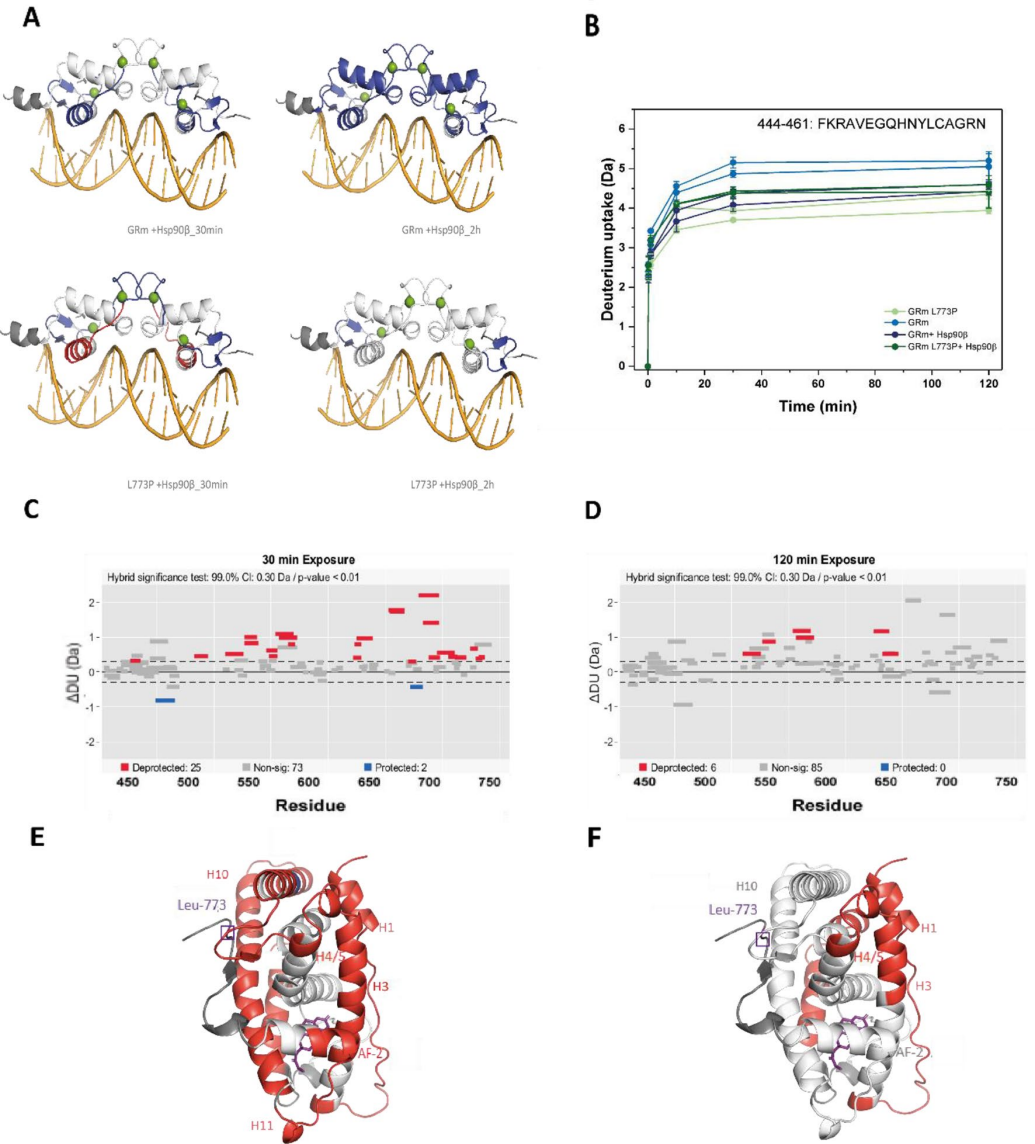
Single point mutation L773P alters the dynamics of several GR elements in the presence of Hsp90β. **(A)** Projection of the differential H/DX data for GRm and GRm L773P in the presence of Hsp90β onto the crystal structure of the GR DBD in complex with an *fkbp5*-derived GRE (PDB ID 3G6R). Significant changes are color coded in blue (low exchange) to red (high exchange). **(B)** Uptake plots for GR residues 444–461 that consist the DNA-reading helix in the absence and presence of Hsp90β. **(C,D)** Wood’s plots based on two experiments constructed with the Deuteros software for time points of 30 and 120 min using hybrid statistics (p-value < 0.01) to compare GRm and GRm L773P in the presence of Hsp90β and ATP. Persisting differences in the presence of Hsp90β concern the H1, H3, H4/5 and H7. **(E,F)** The most significant relative changes in the fractional uptake between the two GR variants that persist in the presence of Hsp90β and ATP are mapped on the GR LBD structure for 30 min and 2 h of exchange and are color coded in blue (low exchange) to red (high exchange).

The differential HD/X data for the two constructs in the presence of Hsp90β and ATP was, therefore, projected onto the structure of dimeric GR DBD in complex with an *fkbp5*-derived GRE (PDB ID 3G6R) to visualize the differences in this domain in the presence of chaperone (Fig. 6A). It can here be observed that the DNA-reading helix in GRm L773P, experiences a deprotection instead of the protection observed for GRm, when Hsp90β is present. After 2 h of exchange, GRmL773P exhibits only minimal changes in this domain compared to GRm, which seems strongly protected. Interestingly, plotting the fractional uptake for the GR DNA-reading helix in the presence and absence of Hsp90β, helps visualize that this element undergoes opposite exchange dynamics in GRm and GRm L773P (as observed in the Wood’s plots in Figs. 4B and 5C) in the presence of chaperone and ultimately, adopts the same exchange status (Fig. 6B).

The comparison of both variants in the presence of the Hsp90β machinery and ATP can help summarize the persistent differences in their interaction with the chaperone, the perturbations that chaperone binding cannot buffer (Fig. 6C–F). This shows that changes originating from elements proximal to the mutation site and the β-sheet 2 of GR, over 2 h of exchange in the presence of Hsp90β and ATP, concern H1, H3, H4/5 and H7. The changes observed in β-sheet 2 and the DBD of the receptor can not be observed in this Wood’s plot, since apparently the chaperone is able to restore their dynamics.

### In silico insight into L773P point mutation conformational consequences

To further understand the conformational consequences of the single-point mutation at position 773, we examined the two GR variants in silico by Molecular Dynamics (MD) simulations in the presence of dexamethasone. We mutated leucine 773 to proline in the FoldX force field, employing the crystal structure solved for GRLBD by Hemmerling et al. and by coloring the protein surface for aminoacid hydrophobicity according to the Kyte-Doolittle scale, it is apparent that there is a very hydrophobic spot around the mutation site that is discontinued once leucine 773 is mutated to proline (Fig. 4E)^37–39^.

**Figure 7:**
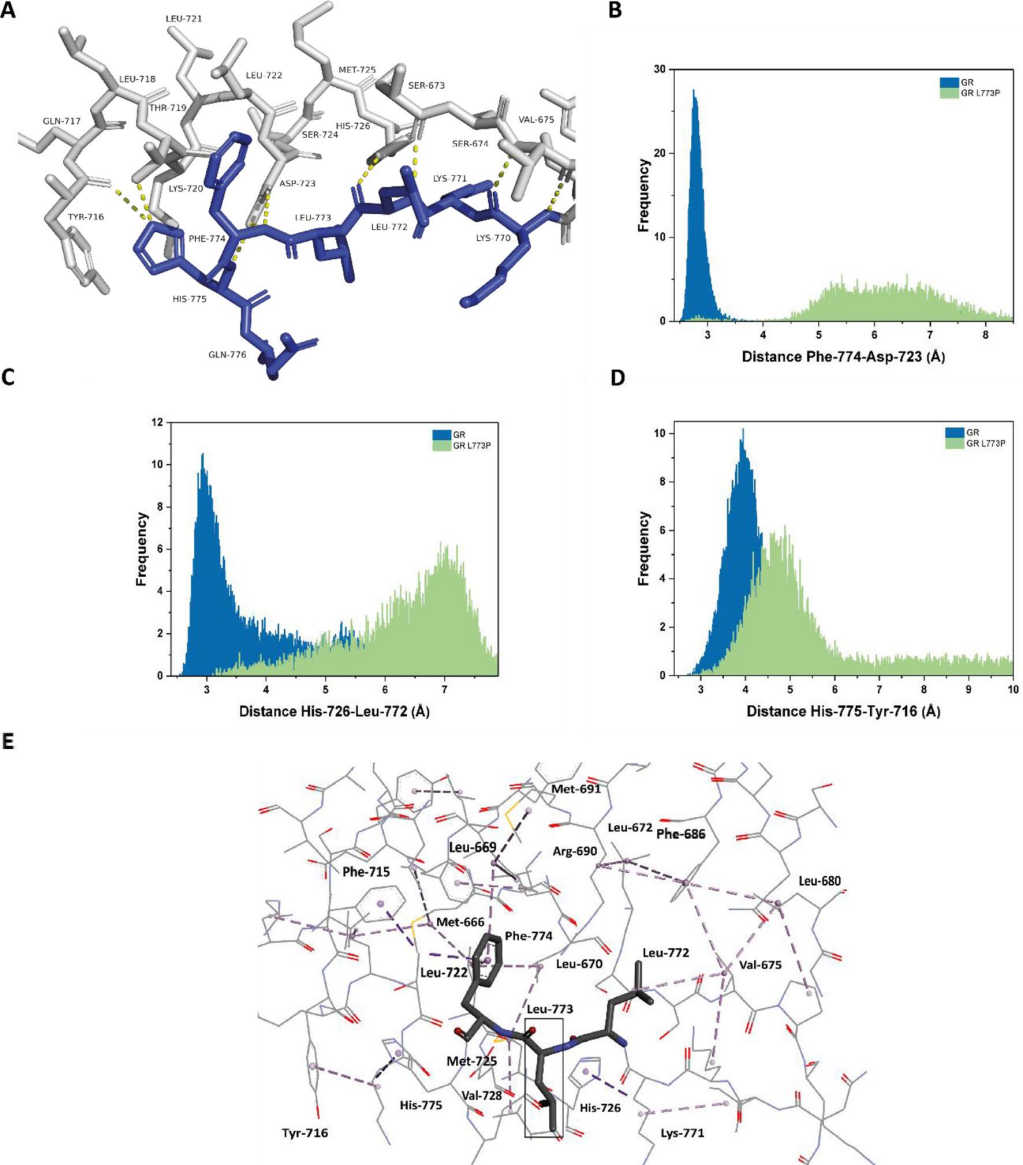
Mutation of leucine 773 to proline leads to disruption of the hydrophobic residue network at GR’s terminus. **(A)** The hydrogen bonding network around leucine 773. **(Β–D)** Hydrogen bond pairs, as indicated in the plots, between GR’s C-terminus and H10 residues exhibit higher distances in GR L773P MD simulations (n = 2, t = 100 ns). **(E)** The hydrophobic interaction network around leucine 773 as visualized with BIOVIA discovery studio shows how the neighboring to the mutation residues, Phe774 and Leu772 are in the epicenter of extensive hydrophobic interactions with GR elements in H10, β-sheet 2, H9 and H8.

With a closer look at the hydrogen bonding network within this hydrophobic spot, it is apparent that the C-terminal residues around the mutation are involved in hydrogen bonds with H10 residues His726, Asp723 and Tyr 716 (Fig. 7A). These residues also show an intense change in the H/DX analysis. We performed MD simulations with the GROMACS software in the CHARMM36 force field to examine hydrogen bonding between the respective pairs^40,41^. The GRm variant displays an average distance of approximately 3 Å between the hydrogen bond donors and acceptors in the MD simulations, a distance consistent with the existence of an H-bond (Fig. 7B–D). In contrast, the mutated construct exhibits considerably larger distances between these pairs, which implies that the point mutation affects the positioning of the C-terminal peptide towards H10 and β-sheet 2. Once hydrophobic interactions are visualized, it is clear that the neighboring to the mutation residues His 775, Phe774, and Leu772, participate in complex hydrophobic interactions, highlighting the importance of proper positioning of GR’s C-terminus (Fig. 7E). The neighboring to the mutation site Phe774 and Leu772 seem to be in the epicenter of hydrophobic interactions with H10 (Tyr 716, His 725, Asp723), β-sheet 2 (Pro676, Val675, Lys770) and also H9 (Leu680, Phe 686, Arg690) and H8 (Leu672) residues that stabilize the other β-strand of β-sheet 2. As other distances, like Leu770-Val675 and His775- Thr719, are not significantly affected in this approach, a local relaxation of the structure, which destabilizes β-sheet 2, is likely the reason for the increased dynamics observed via H/DX in this region (Supplementary Figures S4, S5).

## Discussion

Glucocorticoid receptor is a remarkable molecule, whose exact properties, despite the extensive research, are not yet clarified. We here attempted to gain some insight into the conformational requirements for GR to bind ligand, coactivator and Hsp90β by observing the behavior of a glucocorticoid resistant mutant. Normally, upon agonist binding, a repositioned AF-2 forms a pocket with H3 and H5, forming the lid of the ligand binding pocket^35^. The extended strand following AF-2, where the hGR L773P point mutation addressed in this study resides, seems to be crucial for hormone binding, as truncation of it leads to an inactive receptor^34^. Charmandari et al. report that the mutant receptor demonstrates decreased transcriptional activity but preserves its ability to bind DNA, decreased affinity for ligand, delayed nuclear translocation, altered interaction, only through the AF-1, with the NCOA-2 coactivator and exerts a dominant negative effect on wild-type hGRα in vivo^32^. This behavior is in agreement with the presented in vitro investigations. The L → P mutation at position 773 does not seem to have overt effects on the protein, as despite the slightly lower melting temperature of GRm L773P, both variants are stable up to 40 °C. GRm L773P exhibited a 2.4-fold rate decrease in dexamethasone binding, an altered in vitro interaction of the AF-2 with a NCOA2-derived peptide, and also the Hsp90β chaperone. Moreover, the mutant receptor is competent in DNA binding but with altered dimerization properties.

Transcriptional coactivators develop multiple hydrophobic interactions via their LxxLL helical motifs with the shallow hydrophobic groove that forms between H3, H4 and AF-2 of agonist-bound GR^35,36^. The H3-H5 interaction has previously been reported to act as a switch, conserved among steroid receptors and crucial to receptor sensitivity for ligand^42^. This study introduces a leucine at position 604 of GR’s H5, which may develop a vdW interaction with G567 of H3 and leads to a receptor able to get activated by 10 times lower steroid concentrations. H3 is also affected as a result of the partial unfolding of GR by the chaperone Hsp70, which at the same time causes ligand release^24^. In our previous study, we identified peptides from H3 in crosslinked products with *C.elegans* Hsp90^25^. Importantly, ligand binding residues N564 and Q570 that form three out of six hydrogen bonds between GR and DEX, reside in H3. AF-2 residue L753 is also directly involved in dexamethasone binding^35,36^. H8 has been reported as another important switch for GR function and the L687-690A mutation examined in the respective study, led to decreased transcriptional activity and association of GR with heat shock protein *90 *in vivo, without overt effects on receptor protein stability^43^.

H/DX analysis shows an overall more dynamic GRm L773P, both in the absence and presence of Hsp90β. Significant changes concern H3, H4/5, H8, H9, β-sheet 2, H10, AF-2, and in addition, H1 in the presence of Hsp90β. These regions overlap well with the Hsp90β binding site while hormone binding residues follow intensely altered dynamics, when compared to GRm. Taken together, this data demonstrates that disruption of the hydrophobic network inGRs C-terminus, further translates to an overall perturbation of the extensive conformational changes GR performs upon ligand binding, and besides AF-2, also affects GR elements distant to the mutation site. Importantly, dimerization properties of the receptor are altered, even though the dimer interface is on the opposite site of the molecule (Supplementary Figure S6). Judging from Charmandari and coworkers’ results,the altered dimerization properties, and H/DX behavior of the DBD and hinge regions of GR, it can be expected that the mutant receptor can not meet the conformational requirements for proper domain communication and could therefore, serve as a model to understand the domain interaction of this complex molecule. Hsp90β is thought to maintain the ligand binding pocket in an open state and judging from the extensive deprotection observed by H/DX, it seems probable that the point mutation leads to an overall more exposed pocket. This reflects in the inability of GRm L773P to form a ternary complex with NCOA2 and Hsp90β and would also explain the increased polarization rate of F-DEX while binding to GRm L773P in the presence of Hsp90β: since the two species have the same size, increased polarization should reflect the increased tumbling rate of F-DEX in the deprotected pocket. Hsp90β, however, does restore hormone binding to GRm L773P that coincides with suppression of the H/DX changes observed in the absence of the chaperone. This may be reflecting the buffering effect of Hsp90β on a mutated client protein. There are, however, pronounced differences, in elements overlapping with the GR∙Hsp90β interface, that chaperone binding cannot overcome and at the same time lead to an altered interaction with the Hsp90β chaperone. Especially in the H9- β-sheet 2- H8 and H3-H4/5 interfaces, perturbations persist. Comparing the non-mutated GR construct with GRm L773P supported that proper contacts within the hydrophobic network of residues in GR’s C-terminus are important for allosteric communications in this complex molecule.

## Materials and methods

### Protein expression and purification

Proteins were expressed in Escherichia coli BL21 (DE3) cells, utilizing pET28 plasmids as expression vectors. For His_6_-Hsp90β purification, bacterial cultures were grown at 37 °C to an OD_600_ of 0.6–0.8 and expression was induced by addition of 0.5 mM IPTG. Cells were incubated overnight at room temperature and harvested by centrifugation with 7000 rpm for 15 min at 4 °C. Cell pellets were resuspended in 40 mM HEPES/KOH, 150 mM KCl, pH 7.5 supplemented with protease inhibitor (Serva, Heidelberg, Germany) and DNAseI (Serva, Heidelberg, Germany) and were mechanically disrupted by a hydraulic press (Constant Systems Ltd., Daventry, UK). Cleared lysate was applied onto a HisTrap FF 5 ml column (GE Healthcare, Chicago, USA) and elution was induced by 300 mM imidazole. Protein containing fractions were diluted, applied onto a Resource Q (GE Healthcare, Chicago, USA) column and eluted in a salt gradient. Hsp90 was then purified to homogeneity on a Superdex 200 size-exclusion column (GE Healthcare, Chicago, USA) equilibrated in storage buffer (40 mM HEPES/KOH, 150 mM KCl, 0.5 mM DTT, pH 7.5). GRm, the DBD, hinge and LBD domains of GR stabilized by mutagenesis (F602S/A605V/V702A/E705G/M752T), was expressed at 18 °C overnight in ZYM-5052 media supplied with 250 μM dexamethasone (Serva, Heidelberg, Germany) and purification was performed as described previously^23,44^. Proteins were shock-frozen in liquid nitrogen and stored at − 80 °C. Identity and purity were assessed by MALDI-TOF mass spectrometry (Bruker, Bremen, Germany) and SDS-PAGE (data not shown).

### Analytical ultracentrifugation (AUC)

Sedimentation analysis of F-DEX (Thermo Fischer Scientific, Bremen, Germany) at a concentration of 400 nM, was performed with a ProteomeLab Beckman XL-A analytical ultracentrifuge (Beckman Coulter, Brea CA, USA) with an AVIV fluorescence detection system (Aviv Biomedical Inc., Lakewood CA, USA) as previously described^25^. Ultracentrifugation was carried out at 42,000 rpm at 20 °C in 20 mM HEPES, 20 mM KCl, 5 mM MgCl_2_, pH 7.5. Experiments contained 3 μM of GRm or GRm L773P and 3 μM of the unlabelled chaperones. Nucleotides were added at a concentration of 2 mM. Data analysis was performed by calculating differences between scans from a selected time range and averaging over several of these differentials. The dF/dt data was then normalized against the initial fluorescence intensity. To ensure comparable sample handling, plots were generated from samples measured in the same experiment with automated data processing in the in-house software diffUZ^25,44^. s_20,w_ values were derived from a bi-Gaussian fitting of the dF/dt plots and the error is based on the standard deviation of this approach plus an additional contribution from performing the meniscus picking procedure. Sedimentation velocity experiments with absorbance optics, in which all species are detected without specific labelling were performed with the UV–Vis detector at 280 nm.

### Fluorescence polarization

Hormone-binding kinetics were monitored by fluorescence polarization on a Jasco FP-8500 fluorescence spectrometer (Jasco, Groß-Umstadt, Germany) equipped with polarizers. 1 µM apo-GRm, after extensive dialysis to remove dexamethasone as described, was added to 50 nM fluorescently labelled dexamethasone (F-DEX, Thermo Fischer Scientific, Bremen, Germany)^23,25^. Hsp90β, when present, was added at a concentration of 3 µM. Binding kinetics were recorded at 20 °C in 20 mM HEPES, 20 mM KCl, 5 mM MgCl_2_, 2 mM ATP, pH 7.5. Hormone-binding rates were determined by fitting association kinetics to exponential models and the error bars represent the standard deviation of three independent measurements.

### Fluorescence-labelling of NCOA2-derived peptide

0.05 mg ATTO 488 N-hydroxysuccinimidyl (NHS) ester (ATTO-Tech, Singen, Germany) dissolved in DMSO was added to 0.5 mg of the coactivator-derived peptide (JPT, Berlin, Germany) with a final DMSO concentration of 1%. The reaction was carried out for one hour at room temperature and was quenched with 100 mM Tris, pH 7.5^25,44^. Free label was removed by dialysis against 20 mM Tris, 20 mM NaCl, 5 mM MgCl_2_, pH 7.5 using a Pur-A-Lyzer Mini Dialysis Kit (Sigma, St. Louis, USA) with a 1 kDa molecular weight cut-off.

### Hydrogen deuterium exchange coupled to mass spectrometry (HDX-MS)

Hydrogen/Deuterium exchange mass spectrometry was performed on a fully automated system equipped with a Leap robot (HTS PAL; Leap Technologies, NC), a Waters ACQUITY M-Class UPLC, an H/DX manager (Waters Corp., Milford, MA) and a Synapt G2-S mass spectrometer (Waters Corp., Milford, MA), as described previously^45,46^. 30 μM GRm were diluted 1:20 with deuterium oxide in 25 mM Tris, 100 mM NaCl, 50 μM DEX, pH 7.9) and incubated at 20 °C for 0.17 min, 1 min, 10 min, 30 min, and 2 h. For measurements with the Hsp90 chaperone the protein sample contained 15 μΜ Hsp90 and 15 μΜ GRm and the buffer contained 2 mM ATP. The exchange was quenched by 1:1 dilution with quenching buffer (200 mM Na_2_HPO4, 200 mM NaH_2_PO4, 250 mM Tris (2-carboxyethyl)phosphine, 3 M GdmCl, pH 2.2) at 1 °C. Digestion was carried out a Waters Enzymate BEH Pepsin Column (2.1 × 30 mm) at 20 °C. Peptides were trapped and separated on a Waters AQUITY UPLC BEH C18 column (1.7 µm, 1.0 × 100 mm) with an acetonitrile /H_2_O gradient containing 0.1% (v/v) formic acid at 0 °C to minimize back-exchange. Eluting peptides were directly subjected to the Synapt TOF mass spectrometer by electrospray ionization. Data analysis was conducted with the Waters Protein Lynx Global Server PLGs (version 3.0.3) and DynamX (Version 3.0) software package. We herein present time points 30 min and 2 h, as the earlier time points only describe the emergence of the same signals but on a lower magnitude of exchange. Wood’s plots, applying a hybrid significance test (p-value < 0.01), were generated with the Deuteros software^47^. The respective scripts were exported and used to apply and visualize the color code of the plot on the GR LBD or DBD structure (PDB ID 5NFP and 3G6R respectively) using the PyMOL Molecular Graphics System, Version 2.0 Schrödinger, LLC.

### Atomistic molecular dynamics simulations

Molecular Dynamics simulations were performed on an AMD 3970 × CPU@ 3.7 GHz with the support of a NVIDIA GeForce GTX 1080 graphics chip using GROMACS v2018 and the CHARMM36 force field built in the Linux Ubuntu 18 environment^40,41,48^. Calculations are based on the GRLBD crystal structure with PDB ID 5NFP, solved by Hemmerling et al.^39^ The L773P mutation was generated with FoldX while dexamethasone topology was generated with the CGenFF server^38,49,50^. The Avogadro program was used to assign the dexamethasone hydrogen atom coordinates^51^. The Cgenff_charmm2gmx.py script from the Mac Kerell lab was used to format the ligand topology for GROMACS. The unit cell was defined as a dodecahedron and was solvated in TIP3P water. The protein net charge was neutralized by adding the appropriate Na^+^ and Cl^−^ ions. The energy minimization step and NVT, NPT equilibration steps were performed based on the tutorials and mdp. files provided by Dr. Justin Lemkul (http://www.mdtutorials.com/) with minor modifications^52^. Production MD for data collection was performed for 100 ns (n = 2) and trajectories were analyzed with the GROMACS toolset. Histograms were visualized with Origin 8.6, hydrophobic interactions with BIOVIA discovery studio and Kyte-Doolitle hydrophobicity was colored using UCSF Chimera^37,53^.

### Electrophoretic mobility assays

EMSAs were carried out using Novex TBE Gels, 10% ((Thermo Fischer Scientific, Bremen, Germany). DNA was purchased as forward and reverse oligonucleotides and was hybridized by heating at 98 °C. Electrophoresis was carried out in TAE buffer and as indicated by the manufacturer. After electrophoresis, the gels were stained for 20 min with Clear G DNA stain (Serva, Heidelberg, Germany) in TAE buffer and upon scanning the gel with a Typhoon scanner at green fluorescence to specifically detect DNA, gels were also stained with Coomasie to detect the protein bands.

## Supplementary Information

Supplementary Figures.

## Data Availability

The datasets generated during and/or analysed during the current study are available from the corresponding authors on reasonable request.
