# Supplementary material for: Glucocorticoid resistance conferring mutation in the C-terminus of GR alters the receptor conformational dynamics
Source: Sci Rep. 2021 Jun 15;11:12515. doi: 10.1038/s41598-021-92039-9 (PMC8206104; doi:10.1038/s41598-021-92039-9)
Supplement: Supplementary file 1 — Supplementary Figures. [file 41598_2021_92039_MOESM1_ESM.pdf]

Glucocorticoid resistance conferring mutation in the C-terminus of GR alters the receptor conformational dynamics

Anna Kaziales[1]*, Florian Rührnößl[1] and Klaus Richter[1]*

Supplementary Figures

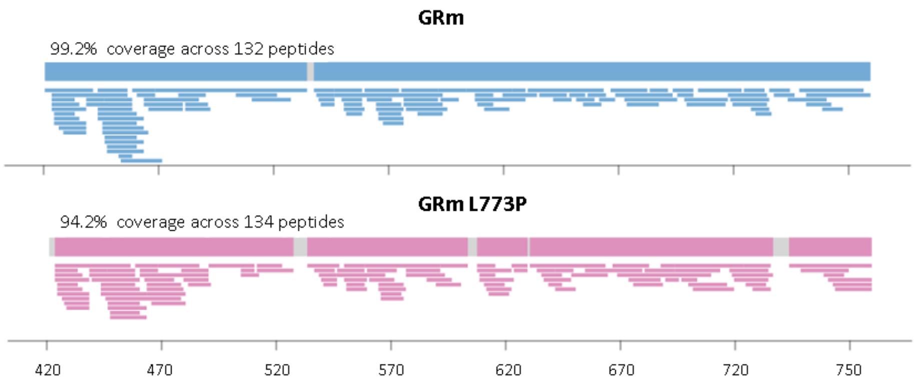

Supplementary Figure S1. Sequence coverage obtained in HD/X MS measurements for the GRm and GRmL773P as visualized by Deuteros.

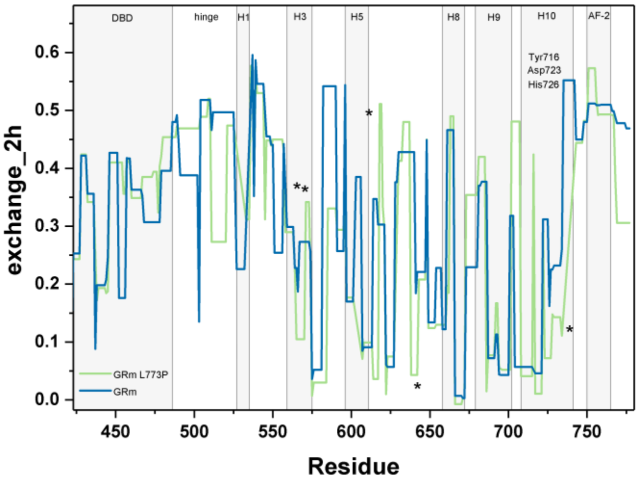

Supplementary Figure S2. The hydrogen-deuterium exchange is plotted for GRm and GRmL773P. Hormone binding residues are indicated with asterisks and characteristic GRLBD elements are highlighted on the plot. Both proteins are highly flexible and differences in their exchange rate can be observed for the various GR elements.

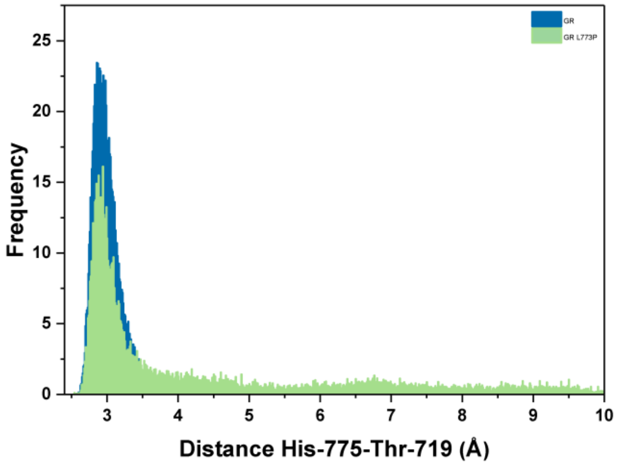

Supplementary Figure S3. Distance calculation of the indicated pair based on MD simulations (n=2, t=100ns).

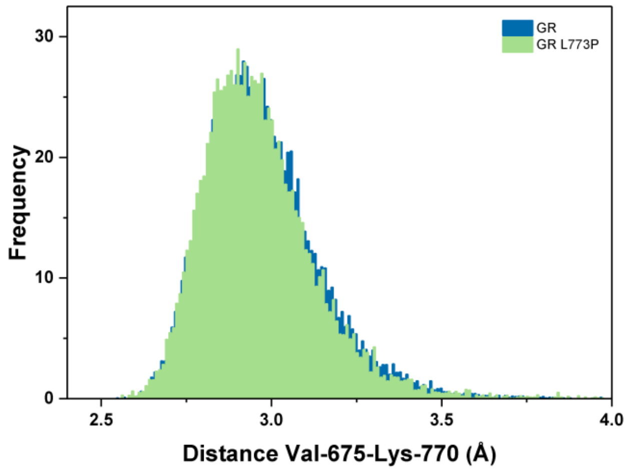

Supplementary Figure S4 Distance calculation of the indicated pair based on MD simulations (n=2, t=100ns).

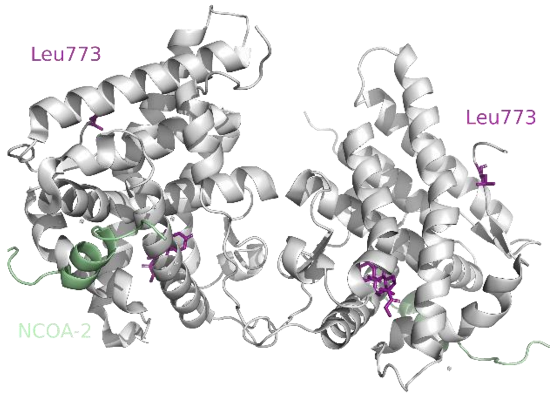

Supplementary Figure S5. Structure of dimeric GR LBD in complex with NCOA-2 peptide and DEX based on PDB ID 1M2Z, indicating the mutation site and the NCOA-2 coactivator binding site.

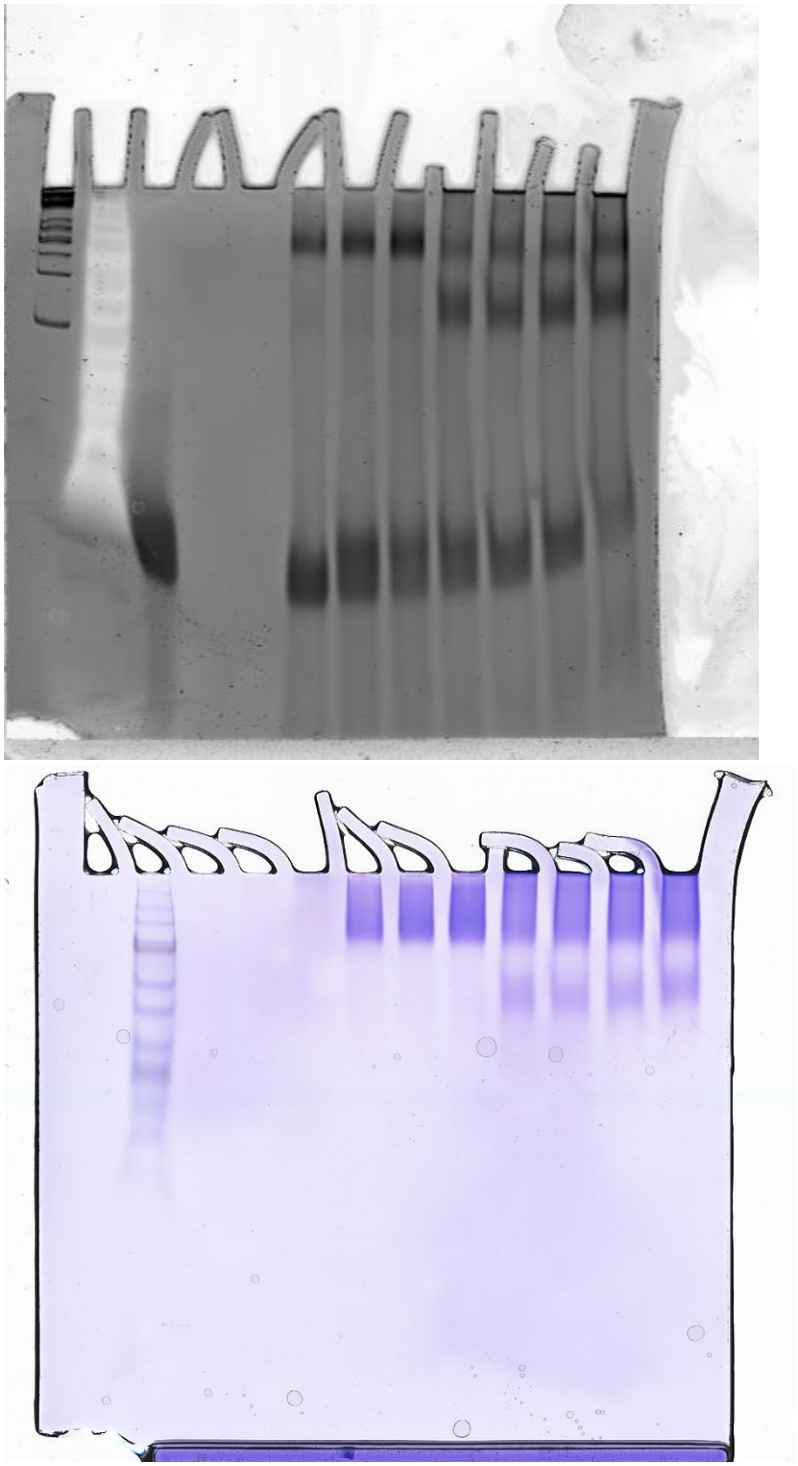

Supplementary Figure S6. Uncropped gel scans corresponding to Figure 3B.
